# Measles surveillance data analysis and serological survey in Quzhou, China, 2014–2024: an assessment of progress toward measles elimination

**DOI:** 10.3389/fmed.2024.1492873

**Published:** 2024-11-05

**Authors:** Xiaoying Gong, Wangfeng Zheng, Shiming Lai, Zhiying Yin

**Affiliations:** ^1^Department of Immunity, Quzhou Center for Disease Control and Prevention, Quzhou, Zhejiang, China; ^2^Department of Orthopedics, Quzhou Hospital of Traditional Chinese Medicine, Quzhou, Zhejiang, China

**Keywords:** measles, elimination, vaccination, surveillance, incidence, antibody

## Abstract

**Background:**

Measles is a disease that can be eliminated through vaccination. In recent years, measles incidence and mortality have been greatly reduced.

**Methods:**

Analyze measles surveillance data from 2014 to 2023 and measles seroepidemiological characteristics of healthy populations in 2024 to assess progress toward measles elimination.

**Results:**

A total of 35 measles cases were reported in the surveillance system from 2014–2023 in Quzhou, with an average annual incidence of 1.6/1 million. Since 2019, the incidence of measles has been lower than 0.5/1 million. A serological survey of 257 healthy people showed that the positive rate of measles IgG antibody was 90.3%, and the immunity of all age groups except 0–5 years old was lower than 95%, which did not reach the threshold of 95% herd immunity required for eliminating measles.

**Conclusion:**

Although the incidence of measles in Quzhou is low, the immunity of healthy people to measles infection is insufficient. Measles is still in the control phase, not in the elimination phase. Inadequate immunity in the population may be due to the failure to achieve ≥95% vaccination coverage and low immunogenicity of the vaccine. Recommends that the quality of routine immunization data be assessed and monitored to verify reported vaccination coverage; at the same time, improve vaccination services and optimize vaccination policies to increase actual vaccination coverage. In addition, it is recommended to adjust the MMR immunization strategy, changing the time of the first MMR vaccination from 8 months of age to 12–15 months of age, and the second dose at 4 to 6 years of age.

## 1 Introduction

Measles is a highly contagious viral disease spread through the respiratory tract that can cause acute complications such as keratoconjunctivitis, otitis media, pneumonia, and encephalitis, and long-term sequelae of subacute sclerosing panencephalitis, and is one of the leading causes of death among young children worldwide (1–3). Measles has sensitive and specific diagnostic tests, and safe and effective vaccines, and humans are the only hosts, making it a potentially eliminable disease (4, 5). The World Health Organization (WHO) set the global goal of measles control and elimination in 1989. In 2012, WHO developed the Global Measles and Rubella Strategic Plan, with the goal to eliminate measles in at least 5 out of 6 regions worldwide by 2020 (6). In 2020, WHO released the Global Measles and Rubella Strategic Framework 2021–2030, which reaffirmed and established the goal of achieving and maintaining regional measles elimination by 2030 (7). The Western Pacific Region, where China is located, developed a Regional Strategy and Action Plan for the Elimination of Measles and Rubella in 2017, urging all member states in the region to eliminate measles (8).

Measles elimination is defined as the absence of endemic measles virus transmission within a designated geographic area for ≥12 months with a good surveillance system in place (8). Sensitive surveillance systems are essential to monitor progress toward measles elimination. China has revised its measles surveillance protocol since 2014, transitioning from case-based surveillance to surveillance for symptoms of acute fever and rash. Epidemiological investigation, sampling, and laboratory confirmation of all reported surveillance cases are conducted to identify circulating measles viruses. Quzhou is a prefecture-level city in eastern China. It covers an area of 8,844 square kilometers and had a permanent population of 2.3 million in 2023. As a local municipal surveillance site, the Quzhou surveillance system meets the quality standards for surveillance set by the WHO every year.

To achieve the elimination goal, immunization coverage needs to be sufficient to achieve and maintain 95% immunity in the population (9). China has been vaccinating children aged 8 months against measles since 1978. Since 2008, measles-rubella (MR) for children aged 8 months and measles, mumps, and rubella (MMR) for children aged 18 months have been included in the immunization program. Since 2018, two doses of MMR have been administered to children aged 8 months and 18 months. In addition to routine immunization, China has carried out several rounds of supplementary immunization activities (SIAs) to further reduce the immunization gap. Since 2010, the Chinese administration has reported annual vaccination coverage of more than 99% for each of the 2 doses of measles-containing vaccine (MCV) (10, 11). The routine immunization coverage of 2 doses of MCV for children aged 8 and 18 months in Quzhou also remains at a high level. Because most measles cases in Zhejiang Province, where Quzhou is located, are unvaccinated young children or adults with unknown immunization history, Zhejiang Province has developed a SIA program for students in the third year of middle school (15 years old) and implemented it throughout the province. High levels of vaccination coverage over many years have led to significant reductions in measles incidence, laying a solid foundation for eliminating measles.

However, in recent years, global vaccination coverage has declined due to vaccine hesitancy and the COVID-19 pandemic. Suboptimal vaccination coverage has led to resurgences and outbreaks of measles, and even to the re-establishment of indigenous transmission in countries where measles had been eliminated (12). China has always had a high annual reporting rate for vaccination, but a 2013 survey assessment of MR vaccination coverage found that administratively reported vaccination coverage may have been overestimated by 5–10 percent (13). This suggests that there may be a discrepancy between administratively reported vaccination coverage and actual coverage. In addition, the first dose of MMR vaccination in China is provided at 8 months of age, and the seroconversion rate of children vaccinated at 8–9 months of age is 80–85% (14), which is lower in immunogenicity compared with the seroconversion of 95% or more in children aged 12 months or older (15). It is generally accepted in many countries that children who receive a dose of MMR before their first birthday should receive two additional doses (one at 12–15 months of age and the other at least 28 days apart). It can be seen that there may be a gap between reported vaccination coverage and seropositivity (2), and vaccination coverage does not necessarily represent the true level of immunity in the population.

Periodic serological surveys can be a useful complementary data point to validate vaccination coverage estimates, and provide a direct measurement of population immunity (8), and are a powerful tool for assessing population immunity and national vaccination policies (16). Serological surveys complement surveillance case data as a way to verify elimination (9). Therefore, we conducted a cross-sectional serological survey of measles immunoglobulin G (IgG) antibodies in 2024. This study analyzed measles surveillance data from 2014 to 2023 and described the serological epidemiological characteristics of measles in the healthy population in 2024 to evaluate the progress of measles elimination and provide a reference for improving the strategy of measles elimination.

## 2 Materials and methods

### 2.1 Data sources

Case data came from the Measles Surveillance System of the Chinese Center for Disease Control and Prevention. The system is national, case- and population-based, and provides laboratory confirmation and guide action. Population data came from the Quzhou Statistics Bureau. Quzhou launched the Child Immunization Information System in 2006. The immunization history of the subjects aged 0–17 years old was obtained from the immunization information system. The immunization history of the subjects aged 18 years and above was determined to be unknown because there was no record in the information system. Vaccination coverage data from the Immunization Information System.

### 2.2 Case definition

Surveillance cases are defined as those with fever, a rash with one of the symptoms of cough, catarrhal rhinitis, conjunctivitis, swollen lymph nodes, arthritis/arthralgia, or suspected by the responsible outbreak reporter for infectious diseases to be measles or rubella. A surveillance case that meets one of the following conditions is a laboratory-confirmed measles case: (1) Those whose blood specimens tested positive for measles IgM antibodies; (2) Pathologic specimens testing positive for measles virus nucleic acid or isolating measles virus; (3) Serum measles IgG antibody titers in the recovery phase are ≥4-fold higher than in the acute phase, or antibody is negative in the acute phase but positive in the recovery phase. All cases in this article are laboratory-confirmed cases.

### 2.3 Serological survey methods

A multi-stage stratified cluster sampling method was used to randomly select 2 counties, and one town in an urban area and one in a rural area from each county were randomly selected to investigate the permanent population aged 0–59 years old according to four groups: 0–5 years old, 6–12 years old, 13–19 years old and 20–59 years old. Obtain information on the sociodemographic characteristics of the subjects through one-on-one questionnaire surveys. The sociodemographic characteristics of subjects aged 0–17 years were obtained from their guardians. Gender representation was required to be balanced when respondents were included. Exclude subjects who have serious illnesses or difficulties in understanding and are unable to cooperate with the research.

### 2.4 Subject informed consent

Based on the principle of informed consent and voluntary participation of subjects. For subjects aged 0–7, their guardian needs to sign an informed consent form; for subjects aged 8–17, they and their guardian need to sign an informed consent form together; for subjects aged 18 and above, they need to sign an informed consent form themselves.

### 2.5 Serological survey sample size

Estimated measles IgG antibody positivity rate of 90% based on available literature (17). According to the sample size formula: N = Z^2^[P(1-P)]/d^2^ × deff. Z was the test statistic, taken as 1.96; P was the antibody positivity rate, taken as 0.9; d was the permissible error, taken as 0.05; deff was the design effect, taken as 1.5; and the estimated sample size was 207. Taking into account that respondents may not have responded, the sample size was increased by 20%, i.e., 248.

### 2.6 Collection and detection of serum samples

3 mL of peripheral venous blood was collected and serum was isolated to detect measles IgG antibody. The enzyme-linked immunosorbent assay (ELISA) was used to detect IgG antibodies with the quantitative detection kit of Institute Virion\Serion GmbH. According to the manufacturer’s instructions, measles virus IgG antibody concentration > 200 mIU/mL is considered positive.

### 2.7 Statistical analysis

Shapiro–Wilk was used to verify that all sample data were non-normal distribution. Measles IgG antibody positivity as categorical variables was summarized as counts and percentages, and Chi-square tests were used for comparison between groups. Antibody concentrations were measured and expressed as M (Q1, Q3). The Mann–Whitney U test was used to compare the antibody concentrations of people of different genders, household registrations, and areas; the Kruskal-Wallis test was used to compare the antibody concentrations of people with different immunization histories and different ages. All data analyses were performed using SPSS 16.0, and *p*-values <0.05 were considered statistically significant, indicating a meaningful difference or association.

## 3 Results

### 3.1 Measles cases in Quzhou

**Figure 1:**
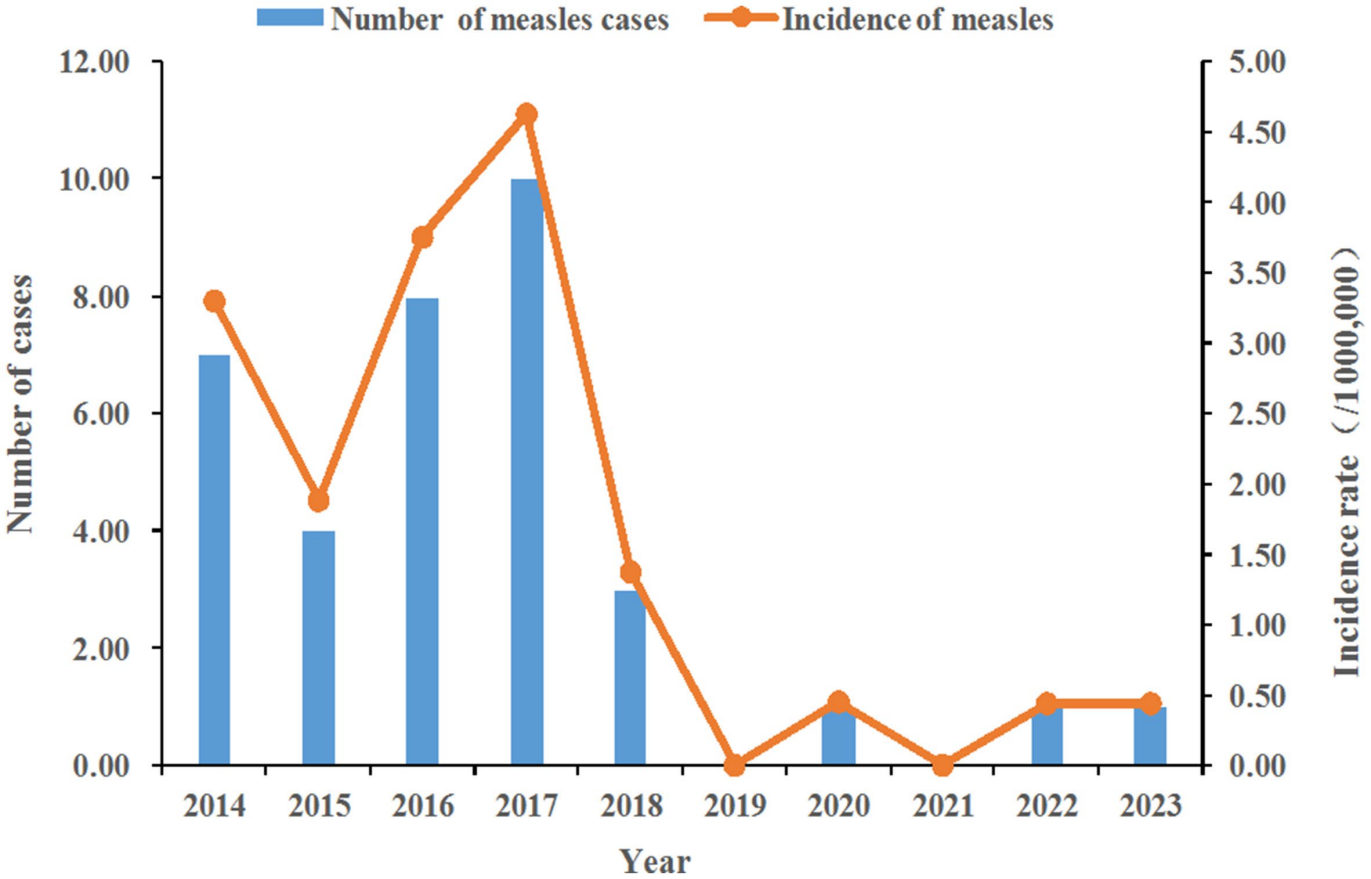
Number and incidence of measles. The figure shows the changes in the number and incidence of measles cases from 2014 to 2023, with measles cases peaking in 2017 and declining significantly after 2019.

A total of 35 cases of measles were reported in the surveillance system from 2014 to 2023, with an average annual incidence of 1.6/1 million. The highest incidence rate was 4.6/1 million (10 cases) in 2017, and the lowest was 0 in 2019 and 2021. Changes in the number and incidence of measles are shown in Figure 1.

Among the 35 cases, 20 were male and 15 were female. The age composition of the onset of the disease was in two groups: 16 cases of preschool children aged 0–5 years (46%) and 19 cases of adults over 27 years of age (54%). Eleven out of 16 preschoolers aged 0–5 years were not vaccinated (69%), 3 received 1 dose (19%) and 2 received 2 doses (12%). The age composition of unvaccinated preschool children was: 4 under 8 months of age (36%), 5 8–23 months of age (46%), and 2 over 2 years of age (18%). Nineteen cases of adults over 27 years of age with unknown immunization history.

### 3.2 Serological cross-sectional survey

#### 3.2.1 Basic information

A total of 257 people were sampled, including 60 (23.35%) people aged 0–5, 71 (27.63%) people aged 6–12, 63 (24.51%) people aged 13–19 and 63 (24.51%) people aged 20–59. The male–female sex ratio was 1:1.2; the proportion of people with household registration inside the city and outside the city was 1:0.2; the proportion of people living in urban and rural areas was 1:1.4. 3% (7/257) had not received MCV, 4% (11/257) had received one dose of MCV, 63% (162/257) had received two or more doses of MCV, 30% (77/257) had an unknown immunization history for MCV.

#### 3.2.2 IgG antibody test results

The measles antibody positivity rate of all subjects was 90.3%, and the median and interquartile range of antibody concentration were 647.1 (373.2, 1179.7) mIU/mL. There was no statistically significant difference in the positive rate (*p* = 0.148) and concentration of measles antibodies (*p* = 0.148) between different genders. There was no statistically significant difference in the positive rate (*p* = 0.548) and concentration of measles antibodies (*p* = 0.543) between different areas. There was no statistically significant difference in the positive rate of measles antibodies among people with different household registrations (*p* = 0.730), but the antibody concentration of people with household registration inside the city was higher than that outside the city (*p* = 0.014), and the difference was statistically significant. There were statistically significant differences in the positive rate (*p* = 0.035) and concentration of measles antibodies (*p*<0.001) among people with different immunization histories. The antibody positivity rate and concentration were higher in the 1-dose group than in the other groups. There was no significant difference in the measles antibody positivity rate among people of different ages (*p* = 0.211), but the antibody concentration in the 0–5-year-old group was higher than that in other groups (*p* < 0.001), and the difference was statistically significant. The data are shown in Table 1.

**Table 1:** The positive rate and concentration of measles antibody in healthy population in Quzhou City.

Variables	Subjects	Antibody positives	Positivity rate	Chi-square	*P*-value	Antibody concentration [mIU/mL, M(Q1,Q3)]	Z/H-value	*P*-value
Genders				2.09	0.148		−1.51*	0.132
Male	119	104	87.39			563.00 (351.40,1102.60)		
Female	138	128	92.75			718.30 (408.60,1264.58)		
Household registration				0.12	0.730		−2.46*	0.014
Inside the city	212	192	90.57			705.30 (414.65,1285.72)		
Outside the city	45	40	88.89			482.10 (328.25,769.85)		
Areas				0.36	0.548		−0.61*	0.543
Urban	107	98	91.59			687.60 (392.50,1223.80)		
Rural	150	134	89.33			613.55 (353.50,1129.85)		
Immunization history				8.62	0.035		25.84**	<0.001
0 dose	7	5	71.43			524.30 (52.10,1900.70)		
1 dose	11	11	100.00			2526.70 (1196.60,3179.40)		
≥2 doses	162	151	93.21			694.60 (444.03,1219.60)		
Unknown	77	65	84.42			540.80 (237.70,861.75)		
Age grouping				4.51	0.211		41.52**	<0.001
Preschool group	60	57	95.00			1379.30 (708.95,2669.98)		
Primary school group	71	61	85.92			533.50 (318.60,819.30)		
Middle school group	63	59	93.65			567.00 (332.20,912.10)		
University and above group	63	55	87.30			554.10 (320.70,930.10)		
Total	257	232	90.27			647.10 (373.15,1179.65)		

*Mann–Whitney U test, **Kruskal-Wallis test.

From 2014 to 2023, the vaccination coverage of MCV routine immunization with 2 doses per year for children in Quzhou was more than 99%, and the MCV-SIA coverage for students in the third year of middle school was more than 95% per year. The data are shown in Table 2.

**Table 2:** The vaccination coverage rate of MCV in Quzhou from 2014 to 2023.

years	MCV1			MCV2			MCV-SIAs*		
	Target population number	Number of vaccinated	Vaccination coverage (%)	Target population number	Number of vaccinated	Vaccination coverage (%)	Target population number	Number of vaccinated	Vaccination coverage (%)
2014	22,373	22,336	99.83	24,925	24,860	99.74	23,892	22,771	95.31
2015	23,511	23,470	99.83	23,255	23,210	99.81	21,153	20,724	97.97
2016	21,257	21,201	99.74	23,807	23,753	99.77	21,477	21,005	97.80
2017	27,940	27,865	99.73	22,021	21,932	99.60	21,497	20,976	97.58
2018	26,083	26,027	99.79	29,530	29,449	99.73	23,027	22,724	98.68
2019	22,481	22,427	99.76	24,524	24,448	99.69	24,003	23,603	98.33
2020	19,735	19,696	99.80	21,835	21,769	99.70	22,187	21,818	98.34
2021	15,606	15,512	99.40	18,831	18,800	99.84	23,153	22,363	96.59
2022	13,535	13,518	99.87	15,177	15,148	99.81	23,045	22,214	96.39
2023	12,201	12,079	99.00	14,063	13,935	99.09	22,003	21,273	96.68

*SIAs for students in the third year of middle school (15 years old).

## 4 Discussion

Measles incidence rates in Quzhou have been below 5/1 million since 2014, and have declined to below 0.5/1 million since 2019, meeting one of the elimination standards set by the Western Pacific region, that is, the incidence of less than 1 confirmed case per million population per year (9). This indicates that the incidence of measles in Quzhou has reached the standard of elimination since 2019. However, serological surveys have shown that the population immunity level has not reached the herd immunity threshold of 95% required for the elimination of measles (15). In the investigated population, except for the 0–5 years old group, the immunity of all other age groups was lower than 95%. Among them, the immunity of 6–12 years old and 20–59 years old is less than 90%. According to the WHO, the elimination of measles epidemiology can be broadly divided into three phases: (1) Similar to pre-vaccination: measles occurs frequently, but epidemics occur every 2–3 years, and the number of cases is inversely proportional to vaccination coverage. (2) Control: Moderate to high vaccination coverage may interrupt measles transmission for some time, resulting in few cases for several years. Eventually, however, the number of susceptible people will gradually increase until there are sufficient numbers to sustain an epidemic. (3) Elimination: Vaccination coverage is sufficient to achieve and maintain 95% population immunity (9). It can be seen that Quzhou is currently in the second phase, the control phase, and although the number of cases has been low for several years, the population immunity does not meet the 95% threshold for herd immunity. Low case numbers may reflect persistently high levels of population immunity and an increased risk of outbreaks, particularly in settings with high vaccination coverage and near elimination, where prolonged periods of low incidence may lead to an accumulation of susceptible individuals (18). When the number (density) of susceptible individuals is large enough, the population is at risk of an outbreak. It has been shown that the resurgence of measles, and even the re-emergence of indigenous transmission in countries where the diseases had been eliminated, may be caused by residual and/or accumulated susceptible populations (8, 19). This suggests that even if surveillance system data meet the incidence criteria for elimination, there is still a risk of measles outbreaks spreading because population immunity is insufficient to achieve and maintain interruption of measles virus transmission. In addition, although the incidence data of the surveillance system is relatively close to the truth, it may be underestimated. Since not all measles patients go to hospitals for treatment and report, cases with milder symptoms may go to convenient community clinics or private clinics compared to crowded and cumbersome hospitals. Most community clinics or private clinics are not as sensitive and standardized in case reporting, so there is a possibility of underreporting. It is recommended to carry out monitoring and evaluation of case reports in community clinics or private clinics to improve the sensitivity of reports.

The significant decrease in measles cases after 2019 may also be related to the COVID-19 epidemic. From 2020 to 2022, Quzhou experienced 3 years of lockdown due to COVID-19. Measures used to slow the spread of COVID-19, such as wearing masks, washing hands, and keeping distance, can also reduce the spread of measles viruses. However, the COVID-19 pandemic has led to severe disruptions in immunization services and reduced vaccination coverage, posing a threat to measles elimination. After the lockdown was lifted in 2023, although vaccination coverage has returned to pre-COVID-19 levels and measles incidence has not increased significantly, the risk of a rebound in the epidemic still exists.

Due to the high seroconversion rate of MCV, almost all people develop immunity after the second dose of MCV (median rate is 97%) (8). Insufficient population immunity to measles is primarily due to failure to achieve the goal of ≥95% vaccination coverage. Although the vaccination coverage rate of the two doses of vaccine in Quzhou is reported to be over 95% or even 99% per year, it is still not enough to achieve and maintain 95% population immunity. This indicates that the actual vaccination coverage rate in Quzhou may be lower than 95%. The target population for administrative reporting coverage estimates generally includes only children enrolled in the jurisdiction as the target population (i.e., the denominator), because it is difficult to capture and include children who are not enrolled in the jurisdiction or who are mobile. Administratively reported vaccination coverage rates tend to be higher than actual coverage rates because the denominator does not adequately include the target population. Relying on reported coverage rather than measured population immunity tends to lead to underestimating the number of susceptible individuals. Routine immunization data quality should be evaluated and monitored to verify reported vaccination coverage using rapid accessibility assessment, batch quality assurance sampling, or 100-household surveys.

Another reason for insufficient immunity in the population may be that China’s strategy of vaccinating with the first dose of MMR at 8 months of age results in low immunogenicity of the vaccine. Due to China’s large, mobile and densely inhabited population, and the high infectiousness of measles, which can easily cause transmission and even outbreaks, China chooses to vaccinate the first dose of MMR at 8 months of age to protect susceptible children whose maternal antibodies decay rapidly. In many countries, the first dose of MMR is usually given between 12 and 15 months of age, and the second dose between 4 and 6 years of age (20). WHO recommends that in countries with high measles prevalence, 9 months is the optimal time to give the first dose of MMR to protect susceptible infants from measles, but this dose does not count as part of the 2-dose regimen (21). The American Academy of Pediatrics and the Committee on Infectious Diseases believe that ensuring that children complete 2 doses of MMR vaccination after their first birthday is critical to measles elimination (22). However, the routine immunization strategy for children in China is to receive only 1 dose of MMR after their first birthday. The incidence of measles in China has now dropped to near elimination levels, and it is recommended to adjust the MMR immunization schedule, like most countries, to adjust the age of the first MMR vaccine from 8 months to 12–15 months, and the second dose to be given at 4 to 6 years of age.

Forty six percent of measles cases were among preschool children aged 0–5 years. Nearly 70% of these cases occurred in children over 8 months old and can be prevented through vaccination. However, due to illness, vaccine contraindications, or delayed or refused vaccination by parents, they missed the opportunity to be vaccinated, resulting in episodes of disease and suboptimal vaccination coverage. Measles is the immunization disease that is most sensitive to vaccination coverage, which also shows that the actual vaccination coverage rate in Quzhou is not very ideal. Measures need to be taken to regularly locate children who have not been vaccinated on time, and to provide services such as reminder, consultation and vaccination. At the same time, parents should be made more aware of the severity of the disease and the impact of vaccination to reduce vaccination hesitancy (23).

A small number of cases have developed despite vaccination, indicating the existence of vaccination failure. Because the vaccine is not 100% effective, as measles vaccination coverage increases, the number of cases among vaccinated people will also increase. Vaccination failure reduces the level of immunity in the population and can lead to the accumulation of susceptible individuals. Vaccination failure is categorized into primary vaccination failure (PVF) and secondary vaccination failure (SVF). PVF has been reported to occur in 10–15% of infants vaccinated at 9 months of age, but 95% of children with PVF gained protective immunity after the second dose (10). Therefore, PVF can be controlled by a second dose of vaccination (24). Studies have shown that although SVF antibody levels are low or even undetectable, they still play an important role due to the presence of an immune recall response (25). SVF cases have a high risk of infection but a low risk of transmission (26, 27). Individuals with SVF are unlikely to pose a threat to measles elimination.

Measles cases reported in the past 10 years were preschool children aged 0–5 years and adults aged 27 years and above. No cases were reported in people aged 6–26 years. This may be because Quzhou conducted multiple rounds of SIAs for children aged 8 months to 14 years between 2004 and 2018, and the annual SIAs for third-year middle school students since 2011, which improved measles immunity in people aged 6–26 years. More than half of cases occurred in adults aged 27 years and older, suggesting that older adults have lower levels of immunity. It is recommended to provide a free policy and establish adult vaccination clinics to vaccinate targeted groups of adults living or working in public Settings (e.g., students, health workers, factory workers, transport and hotel workers, military personnel, police and others entering colleges, universities and workplaces).

In the measles surveillance data, 27 adult measles cases had an unknown immunization history and could not determine whether there was a failure of vaccination. Among the 257 subjects of the serological survey, five of the seven people with no history of immunization (all in the 0–5 age group) were IgG positive, indicating previous measles infection. A total of 173 survey subjects had received at least one dose of measles vaccine. Due to the high seroconversion rate of the measles vaccine, IgG positivity was likely from vaccination. However, the immunization history of 77 survey subjects was unknown, and it was impossible to determine whether IgG positivity was due to vaccination or natural infection. It can be seen that unknown immunization history has a certain impact on the work of eliminating measles. It is recommended to add an adult vaccination module to the immunization program information system and standardize the storage of adult vaccination record files to facilitate the verification of elimination work.

The median concentration of measles antibodies in the 0-5-year-old group was significantly higher than that in other groups, which may be related to the shorter interval between testing and vaccination. It has been observed that seropositive rates and antibody concentrations are higher in populations with a shorter interval since the last vaccination (28). The seropositivity rate decreased with the time of vaccination (29). Those who received 1 dose of vaccine had higher antibody positivity and concentration than those in other groups, also because most of the people who received only 1 dose were children about 1 year old who had not completed 2 doses, and the vaccination time was close to the detection time. The antibody concentration of people with household registration inside the city is higher than that of people outside the city, which may be because people with household registration inside the city are more likely to receive timely and standardized vaccination than those outside the city.

## 5 Conclusion

Although the measles incidence level in Quzhou has reached the elimination standard, the elimination work is still in the control stage because the immunity of the population has not reached the 95% herd immunity threshold required for measles elimination. Inadequate immunity in the population may be due to the failure to achieve ≥95% vaccination coverage and low immunogenicity of the vaccine. Recommends that the quality of routine immunization data be assessed and monitored to verify reported vaccination coverage; at the same time, improve vaccination services and optimize vaccination policies to increase actual vaccination coverage. In addition, it is recommended to adjust the MMR immunization strategy, changing the time of the first MMR vaccination from 8 months of age to 12–15 months of age, and the second dose at 4–6 years of age.

This article has limitations. Our data came from Quzhou and is not representative of China as a whole. More context comparing Quzhou to other regions in China is needed in future studies. However, our research results can provide a reference to adjust the measles vaccination strategies in the future.

## Data Availability

The raw data supporting the conclusions of this article will be made available by the authors, without undue reservation.
